# Peak Lactate During the First Postoperative Day Predicts 90-Day Graft Loss After Liver Transplantation

**DOI:** 10.3390/jcm15072698

**Published:** 2026-04-02

**Authors:** Quirino Lai, Borna Cutic, Licia Iannello, Ivona Hanzek, Fabio Melandro, Robert Baronica, Gianluca Mennini, Vibor Sesa, Massimo Rossi, Anna Mrzljak

**Affiliations:** 1 General Surgery and Organ Transplantation Unit, Department of General and Specialty Surgery, Azienda Ospedaliero-Universitaria Policlinico Umberto I, Sapienza University of Rome, 00161 Rome, Italy; 2 Liver Transplant Center, University Hospital Center Zagreb, 10000 Zagreb, Croatiaanna.mrzljak@kbc-zagreb.hr (A.M.); 3 School of Medicine, University of Zagreb, 10000 Zagreb, Croatia

**Keywords:** liver transplantation, lactate, early allograft dysfunction, graft loss, biomarkers, retransplantation

## Abstract

**Background:** Early identification of liver transplant (LT) recipients at risk of graft failure is crucial to allow timely retransplantation. Lactate levels have been proposed as markers of graft dysfunction, although their optimal timing and predictive value remain uncertain. This study aimed to investigate the prognostic role of early postoperative lactate measurements for predicting 90-day graft loss after LT and compare their performance with established early allograft dysfunction scores. **Methods:** This retrospective observational study included adult patients undergoing their first LT at the Sapienza University of Rome (Italy) between 2013 and 2021, with external validation in an independent cohort from Zagreb University (Croatia) between 2022 and 2025. Lactate levels were evaluated at declamping, at the end of transplantation, and as the peak value within the first postoperative day. Their predictive ability for 90-day graft loss was compared with Early Allograft Dysfunction (EAD) and the Model for Early Allograft Function (MEAF) score using receiver operating characteristic (ROC) analysis. **Results:** A total of 268 LT recipients were analyzed (178 Sapienza and 90 Zagreb). Ninety-day graft loss occurred in 25 (14.0%) patients in Sapienza and 10 patients (11.1%) in Zagreb. A lactate peak within the first postoperative day showed the highest discriminative ability for predicting graft loss in both cohorts (Sapienza: AUC 0.87, 95%CI 0.77–0.98, *p* < 0.001; Zagreb: AUC 0.79, 95%CI 0.62–0.97, *p* = 0.003). This outperformed EAD and MEAF. A lactate peak cutoff of 5.0 mmol/L (75th percentile) resulted in 80.0% sensitivity and 86.3% specificity in Sapienza and 80.0% sensitivity and 68.7% specificity in Zagreb. Higher thresholds increased specificity, reaching 98.7% and 95.0% at 8.4 mmol/L in the Sapienza and Zagreb cohorts, respectively. Patients with a lactate peak ≥ 5.0 mmol/L showed significantly higher 90-day graft loss compared with those below the threshold in both cohorts (Sapienza: 47.6% vs. 4.7%, *p* < 0.001; Zagreb: 25.0% vs. 5.0%, *p* = 0.004). **Conclusions:** The peak lactate value during the first postoperative day represents a simple and accurate biomarker for predicting early graft loss after LT. Its superior predictive performance compared with commonly used EAD-related scores suggests that the lactate peak may represent a valuable tool for early postoperative risk stratification.

## 1. Introduction

Liver transplantation (LT) represents the best therapy for several causes of end-stage liver disease [1]. However, the management of LT patients presents great complexities, and scores able to improve decision-making are extremely relevant, mainly for the early detection of patients needing retransplantation [2]. In recent decades, several scores have been developed to explore the definition of early allograft dysfunction (EAD), with the main focus being to identify cutoffs for retransplantation or futile procedures [3,4,5,6].

In this context, the identification of tools that are easy to calculate vs. complex models is of great relevance [7]. Moreover, the early detection of patients eligible for retransplant is of paramount importance, because earlier diagnosis increases the likelihood of achieving favorable outcomes, avoiding severe infections and adverse events after retransplantation [8,9]. A recent meta-analysis including 17,582 liver transplant recipients identified key predictors of EAD, such as donation after circulatory death, older donor age, higher donor BMI, prolonged cold ischemia time, and increased intraoperative transfusion requirements, with the proposed prediction model showing moderate accuracy for early risk stratification [10]. Another systematic review also identified several potential biomarkers for EAD, including lactate, uric acid, Factor V, and HMGB-1 [11].

In this context, the potential role of a biological parameter for early graft recovery, like lactates, should represent a useful marker to explore and further validate [12,13].

Starting from these considerations, this study aimed to investigate the impact of early (≤one-day) lactate measurements after LT as a predictor of early (≤90-day) graft loss, comparing its role with scores largely adopted for the definition of EAD. This analysis was performed in a single Italian center and validated externally in another European cohort with substantial allocation differences.

## 2. Materials and Methods

### 2.1. Study Design

This retrospective single-center observational study with external validation from another center examined the outcomes of patients receiving their first LT. Approval was obtained from the Local Ethics Boards of Sapienza University of Rome and University Hospital Center Zagreb, and the study followed the Strengthening the Reporting of Observational Studies in Epidemiology (STROBE) guidelines.

### 2.2. Setting and Population

The LT centers at Sapienza University of Rome, AOU Policlinico Umberto I, Rome, Italy, and Zagreb University Center, Zagreb, Croatia, participated in this study. In Rome, a total of 178 LTs were performed during the period 1 January 2013–30 April 2021. In Zagreb, 90 LTs were performed during the period 1 May 2022–31 December 2025. Exclusion criteria for the analysis were retransplantation and pediatric (<16-year-old) transplantation.

### 2.3. Outcomes

The primary outcome was the rate of 90-day graft loss. The last follow-up date was 1 March 2026. All patients had at least 90 days of follow-up; therefore, outcome ascertainment was complete for the primary endpoint.

### 2.4. Data Collection

Data were extracted retrospectively from patient records. Data integrity was overseen by the study group’s Data Manager (QL), who resolved data errors and missing values through queries when feasible.

### 2.5. Definitions

Ninety-day graft loss was defined as a need for retransplantation or patient death due to any cause. EAD was defined according to the Olthoff criteria [3]. MEAF was calculated by adopting the equation reported in the original article in which the score was first proposed [4]. Allocation of grafts followed different rules according to the guidelines of Centro Nazionale Trapianti for Italy and Eurotransplant for Croatia.

### 2.6. Surgical Procedures

Liver transplant procedures were performed by experienced transplant teams at each center, following standardized center-specific protocols. Although minor variations in surgical technique may have occurred over time and between operators, the overall operative approach was consistent within each institution. Liver transplantation was performed using a standard piggyback technique, preserving the recipient’s vena cava, followed by portal, arterial, and biliary reconstruction.

### 2.7. Statistical Analysis

Continuous variables were presented as medians and first–third quartiles (Q1–Q3), while categorical variables were presented as counts and percentages. Categorical comparisons used Fisher’s exact test or chi-square test as appropriate, while the Mann–Whitney U test was used for continuous data. No missing data were observed for the variables explored in the analysis.

The discriminative ability of EAD, the MEAF score, and lactate measurements for the prediction of 90-day graft loss was assessed using receiver operating characteristic (ROC) curve analysis. Lactate measures were as follows: (a) at LT declamping, (b) at the end of transplantation, and (c) from declamping to the end of the first postoperative day (peak value). The area under the ROC curve (AUC) was calculated with corresponding standard errors (SE), 95% confidence intervals (95%CI), and *p*-values testing the null hypothesis of AUC = 0.50. The calculations were separately performed in the two cohorts.

For peak lactate levels during the first postoperative day, predefined percentile-based cutoffs (25th, 50th, 75th, and 90th percentiles) were evaluated in the Sapienza Rome cohort. In addition, an optimal cutoff was identified using the Youden index derived from the ROC curve in the Sapienza Rome cohort. The identified thresholds were separately tested in the Zagreb series. For each threshold, the sensitivity, specificity, positive predictive value (PPV), negative predictive value (NPV), and diagnostic odds ratio (DOR) were calculated. PPV and NPV were derived, accounting for the observed prevalence of the outcome in each cohort. The DOR was computed as follows:

DOR = (Sensitivity × Specificity)/[(1 − Sensitivity) × (1 − Specificity)]

An infinite DOR was reported in cases where sensitivity reached 100%, resulting in a zero denominator.

Given the limited number of events in both the cohorts, a conventional multivariable regression model was considered at high risk of overfitting and was, therefore, not performed. To address this limitation, a penalized regression approach using the least absolute shrinkage and selection operator (LASSO) logistic regression was applied in the Sapienza Rome cohort to identify the most relevant predictors of 90-day graft loss. Candidate variables included a clinically relevant donor, recipient, and intraoperative factors, including lactate measurements. The model derived from the Sapienza cohort was subsequently applied to the independent validation cohort of the Zagreb series without refitting. The odds ratio (OR) and 95% confidence intervals (95%CI) were reported.

To further evaluate the incremental prognostic value of peak lactate within the first 24 h, additional comparative analyses were performed against the established early post-transplant scores EAD and MEAF. For each cohort, logistic regression models were constructed using EAD or MEAF as baseline predictors, and subsequently extended by including peak lactate. Model performance was assessed in terms of discrimination using AUC. Model fit was evaluated using the Akaike information criterion (AIC) and Bayesian information criterion (BIC), with lower values indicating better fit. The incremental value of adding peak lactate was formally tested using the likelihood ratio test comparing nested models.

Kaplan–Meier survival analyses and the log-rank test were used for survival comparisons, with statistical significance set at *p* < 0.05. SPSS version 27.0 (IBM Corp., Armonk, NY, USA) and R software (version 4.5.0, R Foundation for Statistical Computing, Vienna, Austria) were used for analysis.

## 3. Results

The characteristics of the two cohorts are shown in Table 1. The cohort from Rome (n = 178) showed a median follow-up of 92 months (Q1–Q3 = 56–128). During the entire follow-up period, a total of 46/178 (25.8%) graft losses were reported, with 25 (14.0%) cases observed during the first 90 days.

The cohort from Zagreb had a median follow-up of 14 months (Q1–Q3 = 7–28). During the entire follow-up period, a total of 17/90 (18.9%) graft losses were reported, with 10 (11.1%) cases observed during the first 90 days. The specific causes of graft loss in the two cohorts are shown in Supplementary Table S1. Patients with 90-day graft loss in both cohorts exhibited more severe pre-transplant conditions and significantly worse early postoperative graft function, characterized by higher lactate levels, impaired biochemical parameters, and increased rates of EAD and major complications, with minimal impact of donor-related variables (Supplementary Table S2).

Comparing the two cohorts, LT patients in the Zagreb cohort were significantly older compared with Sapienza recipients (median 60 vs. 57 years, *p* < 0.001). The waiting list duration was longer in Sapienza (4.7 vs. 2.5 months, *p* = 0.005). MELD scores were comparable between groups (*p* = 0.34), whereas MELDNa was significantly higher in Zagreb (21 vs. 17, *p* = 0.006). The etiological distribution differed significantly. HCV-related cirrhosis (32.0% vs. 4.4%, *p* < 0.001), HBV-related cirrhosis (18.0% vs. 5.6%, *p* = 0.005), MASLD-related cirrhosis (14.6% vs. 2.2%, *p* = 0.001), and acute liver failure (11.2% vs. 2.2%, *p* = 0.009) were more frequent in Sapienza. Conversely, alcohol-related cirrhosis was more prevalent in Zagreb (54.4% vs. 41.0%, *p* = 0.04).

Cold ischemia time (CIT) and warm ischemia time (WIT) were significantly longer in Sapienza (400 vs. 291 min and 63 vs. 47 min, respectively; both *p* < 0.001).

Lactate levels at declamping were higher in Zagreb (4.3 vs. 3.5 mmol/L, *p* < 0.001), whereas lactates at the end of LT and on post-LT Day 1 were higher in Sapienza (both *p* ≤ 0.001). Peak lactate within post-LT Day 1 was higher in Zagreb (4.4 vs. 3.7 mmol/L, *p* < 0.001).

Peak AST and ALT within post-LT Day 3 did not differ significantly between centers. However, the bilirubin peak within post-LT Day 3 and the bilirubin on post-LT Day 7 were markedly higher in Sapienza (both *p* < 0.001). INR values were also significantly higher in Sapienza at Day 7 (*p* < 0.001).

Early allograft dysfunction (EAD) was more frequent in Sapienza (37.1% vs. 20.0%, *p* = 0.005). MEAF scores were significantly higher in Sapienza (median 4.3 vs. 3.0, *p* < 0.001), and MEAF ≥ 5 occurred more often (35.4% vs. 13.3%, *p* < 0.001). ICU stay and total hospital length of stay were significantly longer in Sapienza (both *p* < 0.001). Conversely, major complications (Clavien-Dindo ≥ 3a) were more frequent in Zagreb (52.2% vs. 29.2%, *p* < 0.001).

The donors in Zagreb were older (63 vs. 57 years, *p* = 0.03) and had a higher BMI (28 vs. 26 kg/m^2^, *p* < 0.001). Causes of donor death were similar overall, although uncommon causes (i.e., abscesses or cerebral tumors) were more frequent in Zagreb (*p* = 0.02). Donor ICU stay duration did not differ significantly.

### 3.1. Diagnostic Performance Analysis of Lactates

In the Sapienza cohort, peak lactate within post-LT Day 1 showed excellent discriminatory ability (AUC 0.87, 95%CI = 0.77–0.98, *p* < 0.001), outperforming EAD (AUC 0.56, *p* = 0.31) and MEAF (AUC 0.58, *p* = 0.20). In Zagreb, peak lactate also demonstrated good performance (AUC 0.79, 95%CI = 0.62–0.97, *p* = 0.003), whereas EAD and MEAF both showed moderate discrimination (AUC 0.73, *p* = 0.02).

Lactate values at declamping and at the end of LT showed limited or inconsistent predictive performance between centers (Table 2).

External calibration analysis in the Zagreb cohort showed that peak lactate within the first postoperative day had the lowest Brier score (0.089), indicating the best overall predictive accuracy among the evaluated models. Although the Hosmer–Lemeshow test for peak lactate was significant (*p* = 0.009), this result should be interpreted with caution, given the limited number of events in the validation cohort and the known instability of this test in small samples (Table 2).

Analyzing the different thresholds of peak lactate within post-LT Day 1 in the Rome Sapienza cohort, at the 75th percentile cutoff (5.0 mmol/L), peak lactate achieved a sensitivity of 80.0% and specificity of 86.3% in Sapienza (DOR 25.20). In Zagreb, the same value provided 80.0% sensitivity and 68.7% specificity (DOR 8.78).

At the 90th percentile (8.4 mmol/L), specificity markedly increased in both cohorts (98.7% in Sapienza; 95.0% in Zagreb), with high diagnostic odds ratios (161.34 and 19.00, respectively) at the expense of sensitivity (68.0% and 50.0%).

The optimal cutoff identified using the Youden index was 5.6 mmol/L, corresponding to an intermediate threshold between the 75th and 90th percentiles and supporting the robustness of the predefined percentile-based approach.

Lower percentile thresholds improved sensitivity and negative predictive value, particularly at the 25th percentile, where NPV exceeded 95% in both cohorts (Table 2).

### 3.2. Multivariable Models Incorporating Lactates

In the Sapienza cohort, a parsimonious multivariable logistic regression model was generated. After selecting the most relevant variables with the LASSO method, peak lactates within the first 24 h after LT remained independently associated with 90-day graft loss (OR = 1.73, 95%CI = 1.34–2.25, *p* < 0.001), while MELD-Na retained a weaker but statistically significant association (OR = 1.07, 95%CI = 1.00–1.14, *p* = 0.041). When the model was applied to the external validation cohort of Zagreb without refitting, discrimination remained acceptable, with an AUC of 0.79 (95%CI = 0.57–0.97). When the same variables were re-entered into the multivariable model of the Zagreb cohort, peak lactate within the first 24 h remained significantly associated with 90-day graft loss (OR = 1.55, 95%CI = 1.21–1.98, *p* < 0.001) (Table 3).

We also performed additional comparative analyses to assess the incremental prognostic value of peak lactate over established early post-transplant scores. In both cohorts, the addition of peak lactate within the first 24 h significantly improved the performance of models based on either EAD or MEAF, as shown by higher AUC values, lower AIC/BIC, and significant likelihood ratio tests. In the Sapienza cohort, adding peak lactate increased the AUC from 0.56 to 0.87 for EAD-based models and from 0.58 to 0.87 for MEAF-based models. In the Zagreb cohort, the corresponding AUCs increased from 0.73 to 0.81 and from 0.73 to 0.78, respectively (Table 4).

### 3.3. Graft Loss According to Lactate Peak Cutoffs

**Figure 1:**
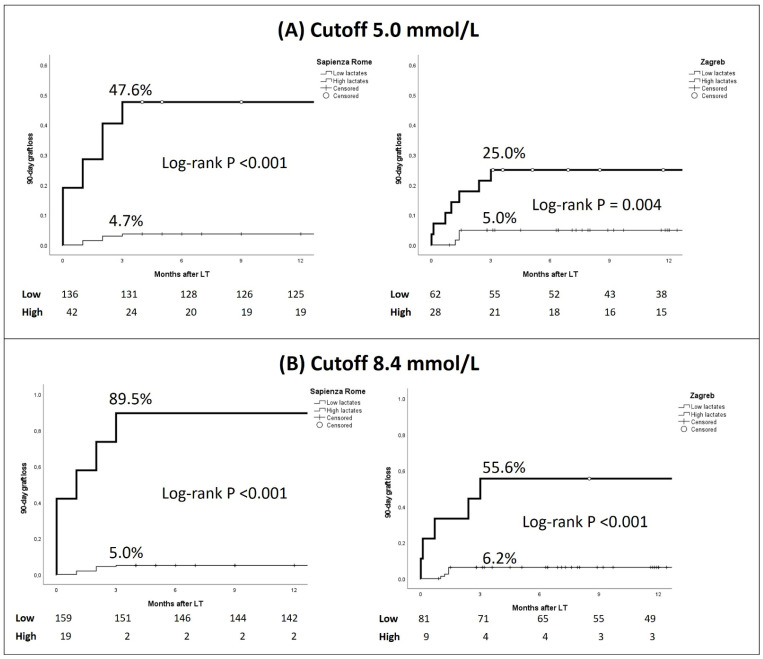
Ninety-day graft loss according to peak lactate levels during the first postoperative day after liver transplantation. (**A**) Cutoff 5.0 mmol/L; (**B**) cutoff 8.4 mmol/L.

Kaplan–Meier analysis demonstrated a strong association between the post-LT Day 1 peak lactate and 90-day graft loss in both cohorts (Figure 1). Using the 5.0 mmol/L threshold, patients with high lactate levels showed significantly higher 90-day graft loss compared with those below the cutoff in both centers. In the Sapienza cohort, 90-day graft loss was 47.6% in the high-lactate group versus 4.7% in the low-lactate group (log-rank *p* < 0.001). In the Zagreb cohort, graft loss occurred in 25.0% of patients with lactate ≥ 5.0 mmol/L compared with 5.0% in those below the threshold (log-rank *p* = 0.004).

**Figure 2:**
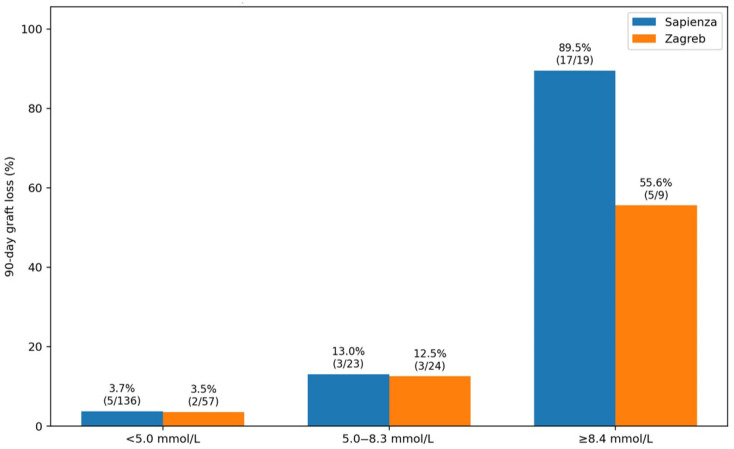
Clinical risk stratification for 90-day graft loss based on peak lactate within the first postoperative day.

When applying a higher threshold of 8.4 mmol/L, the discriminatory capacity further increased. In Sapienza, 90-day graft loss reached 89.5% in patients with lactate ≥ 8.4 mmol/L, compared with only 5.0% in patients with values below this cutoff (log-rank *p* < 0.001). Similarly, in Zagreb, graft loss was 55.6% above the 8.4 mmol/L cutoff versus 6.2% below it (log-rank *p* < 0.001) (Figure 2).

## 4. Discussion

The results of the present study confirmed the relevance of lactate measurement as a useful tool for the prediction of early graft loss after LT. According to the observed results, the peak value reported during the first 24 h from LT declamping was the best tool for predicting graft loss, which is superior to conventionally adopted predictors of early graft dysfunction like EAD and MEAF scores. Such an ability was observed in two different contexts, namely the Italian Rome Sapienza cohort and the Croatian Zagreb series, underlying the relevance of lactates despite differences in donor–recipient matching, overall graft quality, and allocation differences.

The results reported in the present study are in line with other studies previously published.

A single-center retrospective study from the United States explored 1067 adult LT recipients transplanted between 2012 and 2023, evaluating intraoperative lactate kinetics and their association with post-transplant mortality. A time-weighted average lactate cutoff of 3.1 mmol/L predicted 1-year mortality (AUC 0.64, 95%CI 0.57–0.71). Higher intraoperative lactate values were associated with worse survival at 30 days, 1 year, and 3 years (all *p* ≤ 0.01). Lactate clearance between reperfusion and the end of surgery was independently associated with 30-day mortality (hazard ratio = 0.69, 95%CI = 0.60–0.80) [12].

Another single-center retrospective cohort from the United States explored 989 adult LT recipients transplanted during the period 2002–2013. The first lactate level on ICU arrival post-LT was higher in non-survivors (30-day death vs. alive: 3.8 vs. 2.3 mmol/L, *p* < 0.001; in-hospital death vs. alive: 3.7 vs. 2.3 mmol/L, *p* < 0.001) [13].

A single-center retrospective study from Belgium explored 226 deceased-donor LT patients during the period 2013–2019, reporting that the lactate values measured at ICU admission immediately after LT were able to discriminate 1-year mortality (cutoff: 2.25 mmol/L, sensitivity 0.71, specificity 0.72), 30-day mortality (cutoff: 2.65 mmol/L, ROC 0.91, 95%CI = 0.84–0.97), and major postoperative complications (cutoff: 2.55 mmol/L, ROC 0.63, 95%CI 0.56–0.71) [14].

A single-center study from Italy explored postreperfusion lactate clearance after six hours in 70 LT patients. Perioperative lactate kinetics predicted early graft recovery, with an ROC AUC of 0.83 (clearance cutoff: 59.7%, sensitivity 90%, specificity 38.3%) [15].

In the setting of transplantation, the role of lactate clearance has recently regained popularity due to the use of normothermic perfusion machines, confirming the immediate physiological relevance of its decline during the graft function restoration [16].

A multicenter study across six high-volume LT centers reported 509 livers transplanted after normothermic perfusion. A correlation between perfusate lactates measured at different time points and MEAF was reported [17].

Apart from the transplant setting, lactates have been largely explored in the setting of cirrhosis and acute liver failure. A single-center study focused on 133 critically ill cirrhosis patients admitted to the ICU during the period 2011–2016 and proposed a score combining MELD and lactates. The MELD-LA (AUC = 0.89) was superior to APACHE-IV and CLIF-C ACLF for the risk of ICU mortality [18].

Apart from providing confirmatory information, the present study provides several novel insights into the field of early graft dysfunction assessment after LT. First, while previous studies mainly focused on intraoperative lactate levels or single postoperative measurements, our analysis specifically explored different time points, particularly focusing on the peak lactate value during the first postoperative day. The present analysis demonstrated that this parameter represented the most accurate predictor of early graft loss. Second, the study directly compared lactate measurements with widely adopted EAD-related scores such as EAD and MEAF, showing the clearly superior discriminative ability of peak lactates. Finally, the analysis was externally validated in an independent European cohort characterized by different allocation policies and donor–recipient matching, confirming the robustness of the observed association.

From a clinical perspective, the identification of a simple and rapidly available biomarker, such as lactate peak during the first postoperative day, may have relevant implications. Unlike complex prognostic models requiring multiple parameters collected over several days [5,6], lactate measurement is routinely available and can be easily integrated into early postoperative monitoring. Early identification of patients at high risk of graft failure may facilitate timely listing for retransplantation, which is known to improve outcomes when performed before the development of severe complications [9].

From a practical standpoint, the identification of clinically meaningful thresholds allows direct translation of these findings into bedside decision-making. Patients with peak lactate values below 5.0 mmol/L can be considered at low risk of early graft loss, whereas values between 5.0 and 8.4 mmol/L identify an intermediate-risk group requiring closer monitoring. Conversely, lactate levels ≥ 8.4 mmol/L define a high-risk subgroup with a markedly increased probability of early graft failure, in whom early multidisciplinary evaluation and consideration for retransplantation may be warranted.

Importantly, lactate peaks within the first postoperative day provide this information significantly earlier than conventional EAD-based scores, which typically require several days of observation. This earlier risk stratification may allow clinicians to anticipate clinical deterioration and optimize timing for retransplant listing, potentially improving patient outcomes.

Several limitations of the present study should be acknowledged. First, the analysis was retrospective and, therefore, subject to the inherent biases of observational studies. Second, although an external validation cohort was included, the overall sample size remained relatively limited, particularly with regard to the number of early graft losses. Third, the two cohorts differed in several donor- and recipient-related characteristics as well as in allocation policies, which could potentially influence postoperative lactate dynamics. Nevertheless, the consistency of the results observed across the two centers supports the robustness of the findings. Fourth, complex models like L-Graft and EASE were not available for calculation due to the complexity of retrospectively obtaining all the information required for their calculation. A further limitation of this study was the lack of uniformly available detailed donor information, including CMV and viral hepatitis status, across the two cohorts, which precluded their inclusion in the analysis and may have introduced residual confounding. Another limitation was the lack of standardized and granular surgeon-level data (e.g., the number of surgeons involved per procedure or detailed technical variations), which were not uniformly recorded across the two centers. Although all procedures were performed by experienced transplant teams using standardized protocols, we cannot exclude the potential impact of unmeasured procedure-related factors on outcomes. Finally, lactate levels may be influenced by several intraoperative factors, including hemodynamic instability, transfusion requirements, and graft ischemia–reperfusion injury, which could not be completely controlled in the present analysis.

In conclusion, the peak lactate value during the first postoperative day may represent a simple and effective biomarker for the prediction of early graft loss after LT. Its superior discriminative ability compared with commonly adopted EAD-related scores suggests that peak lactate may represent a valuable tool for early postoperative risk stratification. Further multicenter studies are warranted to confirm these findings and to explore the potential integration of lactate-based parameters into future prognostic models for LT recipients.

## Figures and Tables

**Table 1:** Characteristics of the Sapienza Rome and Zagreb cohorts.

Variables	Sapienza Rome (n = 178)	Zagreb (n = 90)	*p*
	Median (Q1–Q3) or n (%)		
**Patient-related variables**			
Age, years	57 (48–62)	60 (55–65)	<0.001
Male sex	150 (84.3)	69 (76.7)	0.14
Caucasian	172 (96.6)	90 (100.0)	0.18
BMI	27 (24–29)	26 (23–29)	0.94
WL duration, months	4.7 (0.9–8.5)	2.5 (0.8–5.5)	0.005
HCC positivity	87 (48.9)	32 (35.6)	0.051
HCV-related cirrhosis *	57 (32.0)	4 (4.4)	<0.001
HBV-related cirrhosis *	32 (18.0)	5 (5.6)	0.005
Alcohol-related cirrhosis *	73 (41.0)	49 (54.4)	0.04
MASLD-related cirrhosis *	26 (14.6)	2 (2.2)	0.001
ALF *	20 (11.2)	2 (2.2)	0.009
Other disease as indication for LT *	15 (8.4)	15 (16.7)	0.06
MELD	16 (11–23)	17 (11–26)	0.34
MELD Na	17 (10–26)	21 (12–30)	0.006
**Transplantation-related variables**			
CIT, minutes	400 (390–425)	291 (219–382)	<0.001
WIT, minutes	63 (58–68)	47 (39–68)	<0.001
Lactates at LT declamping, mmol/L	3.5 (2.8–4.0)	4.3 (3.4–5.0)	<0.001
Lactates at end of LT, mmol/L	2.4 (1.8–3.4)	1.8 (1.0–3.0)	<0.001
Lactates at 1 day after LT, mmol/L	1.5 (1.1–2.1)	1.1 (0.8–1.6)	0.001
Peak lactates within post-LT Day 1, mmol/L	3.7 (2.9–4.8)	4.4 (3.4–5.4)	<0.001
AST peak at post-LT Day 3, IU/L	867 (493–1470)	849 (526–1626)	0.51
ALT peak at post-LT Day 3, IU/L	590 (366–1163)	532 (263–1045)	0.21
Bilirubin peak at post-LT Day 3, mg/dL	2.4 (2.2–7.0)	0.6 (0.4–1.7)	<0.001
INR peak at post-LT Day 3	1.41 (1.29–1.59)	1.50 (1.30–1.76)	0.041
Bilirubin on post-LT Day 7, mg/dL	7.0 (3.8–11.9)	0.6 (0.3–1.1)	<0.001
INR on post-LT Day 7	1.20 (1.11–1.32)	1.06 (1.00–1.15)	<0.001
EAD	66 (37.1)	18 (20.0)	0.005
MEAF	4.3 (3.1–5.8)	3.0 (1.5–4.0)	<0.001
MEAF ≥ 5	63 (35.4)	12 (13.3)	<0.001
ICU stay, days	8 (5–16)	3 (2–5)	<0.001
Length of stay, days	19 (16–32)	15 (11–29)	<0.001
Clavien-Dindo ≥ 3a	52 (29.2)	47 (52.2)	<0.001
**Donor-related variables**			
Age, years	57 (46–67)	63 (53–70)	0.03
Male sex	90 (50.6)	50 (55.6)	0.52
Trauma as cause of death	45 (25.3)	27 (30.0)	0.47
Anoxia as cause of death	3 (1.7)	6 (6.7)	0.07
Cerebrovascular accident as cause of death	125 (70.2)	58 (64.4)	0.40
Other condition as cause of death	1 (0.6)	5 (5.6)	0.02
ICU stay, days	4 (3–5)	4 (3–7)	0.11
BMI	26 (24–28)	28 (24–31)	<0.001

* Some patients presented multiple causes of liver disease contemporaneously. Variables: Q1, 25% quartile; Q3, 75% quartile; BMI, body mass index; WL, waiting list; HCC, hepatocellular carcinoma; HCV, hepatitis C virus; HBV, hepatitis B virus; MASLD, metabolic dysfunction-associated steatotic liver disease; ALF, acute liver failure; LT, liver transplantation: MELD, model for end-stage liver disease; Na, sodium; CIT, cold ischemia time; WIT, warm ischemia time; AST, aspartate aminotransferase; ALT, alanine aminotransferase; INR, international normalized ratio; EAD, early allograft dysfunction; ICU, intensive care unit.

**Table 2:** Predictive performance of lactate measurements, EAD, and MEAF for 90-day graft loss after liver transplantation; external calibration performance of predictive models in the Zagreb cohort; and diagnostic accuracy of lactate peak thresholds during the first postoperative day.

Time	AUC	SE	*p*	95%CI		AUC	SE	*p*	95%CI	
				Lower	Upper				Lower	Upper
**Sapienza**						**Zagreb**				
EAD	0.56	0.06	0.31	0.44	0.69	0.73	0.10	0.02	0.54	0.91
MEAF	0.58	0.06	0.20	0.46	0.70	0.73	0.12	0.02	0.50	0.96
Lactates at declamping	0.60	0.06	0.12	0.47	0.72	0.54	0.10	0.67	0.36	0.73
Lactates at end LT	0.58	0.05	0.19	0.48	0.69	0.73	0.09	0.02	0.57	0.90
Lactates peak on 1st day	0.87	0.05	<0.001	0.77	0.98	0.79	0.09	0.003	0.62	0.97
						**Brier Score**		**Hosmer–Lemeshow *p*-Value**		
EAD	**-**					0.094		0.89		
MEAF	**-**					0.092		0.11		
Lactates at declamping	**-**					0.121		0.54		
Lactates at end LT	**-**					0.097		0.54		
Lactates peak on 1st day	**-**					0.089		0.009		
**Lactates Peak On 1st Day**	**Sens%**	**Spec%**	**PPV%**	**NPV%**	**DOR**	**Sens%**	**Spec%**	**PPV%**	**NPV%**	**DOR**
**Sapienza**						**Zagreb**				
25 centile (2.9 mmol/L)	92.0	26.8	17.0	95.4	4.21	100.0	8.7	12.0	100.0%	∞
50 centile (3.7 mmol/L)	92.0	52.9	24.1	97.6	12.92	90.0	28.7	13.6	95.9	3.63
75 centile (5.0 mmol/L)	80.0	86.3	48.7	96.4	25.20	80.0	68.7	24.2	96.5	8.78
Youden (5.6 mmol/L)	74.0	92.0	64.5	96.0	32.7	70.0	82.0	38.0	95.5	10.6
90 centile (8.4 mmol/L)	68.0	98.7	89.5	95.0	161.34	50.0	95.0	55.5	93.9	19.00

Abbreviations: AUC, Area Under the Receiver Operating Characteristic Curve; SE, Standard Error; CI, Confidence Interval; EAD, Early Allograft Dysfunction; MEAF, Model for Early Allograft Function; LT, Liver Transplantation; Sens, Sensitivity; Spec, Specificity; PPV, Positive Predictive Value; NPV, Negative Predictive Value; DOR, Diagnostic Odds Ratio.

**Table 3:** Multivariable logistic regression analysis for 90-day graft loss in the Sapienza and Zagreb cohorts.

Variables	Beta	SE	OR	95% CI	*p*-Value
**Sapienza**					
Lactate peak at 24 h	0.55	0.13	1.73	1.34–2.25	<0.001
MELD-NA	0.07	0.03	1.07	1.00–1.14	0.04
ALF	0.93	0.75	2.53	0.58–11.09	0.22
**Zagreb**					
Lactate peak at 24 h	0.44	0.12	1.55	1.21–1.98	<0.001
ALF	2.70	1.59	14.87	0.66–336.19	0.09
MELD-NA	0.01	0.04	1.01	0.93–1.09	0.88

Variables initially tested with the LASSO method: patient-related (male sex, BMI, waiting list duration in months, age, HCC, HCV, HBV, alcohol, MASLD, ALF, and MELD-NA), transplant-related (CIT, WIT, AST peak ≤ 3 days, ALT peak ≤ 3 days, bilirubin ≤ 3 days, bilirubin at Day 7, INR peak ≤ 3 days, INR Day 7, and peak lactates ≤ 24 h), and donor-related methods (national share, age, male sex, days in intensive care unit, BMI, cause of death: trauma, anoxia, and cerebrovascular accident). Abbreviations: SE, standard error; OR, odds ratio; CI, confidence intervals; MELD-NA, model for end-stage liver disease Sodium; ALF, acute liver failure; LASSO, least absolute shrinkage and selection operator; BMI, body mass index; HCC, hepatocellular carcinoma; HCV, hepatitis C virus; HBV, hepatitis B virus; MASLD, metabolic dysfunction-associated steatotic liver disease; CIT, cold ischemia time; WIT, warm ischemia time; AST, aspartate aminotransferase; ALT, alanine aminotransferase; INR, international normalized ratio.

**Table 4:** Incremental prognostic value of peak lactate over EAD and MEAF for the prediction of 90-day graft loss.

Cohort	Base Model	AUC	+Lactates 24 h	ΔAUC	BaseAIC	+Lactates 24 h	Base BIC	+Lactates 24 h	LR Test *p*-Value
Sapienza	EAD	0.56	0.87	+0.30	147.0	98.9	153.4	108.5	<0.001
	MEAF	0.58	0.87	+0.29	146.7	99.3	153.1	108.8	<0.001
Zagreb	EAD	0.73	0.81	+0.08	57.8	47.8	62.8	55.3	0.001
	MEAF	0.73	0.78	+0.05	56.4	49.2	61.4	56.7	0.002

Abbreviations: AUC, area under the curve; Δ, delta; AIC, Akaike information criterion; BIC, Bayesian information criterion; LR, likelihood ratio; EAD, early allograft dysfunction; MEAF, model for early allograft function.

## Data Availability

The original contributions presented in this study are included in the article/Supplementary Materials. Further inquiries can be directed to the corresponding author.

## Supplementary Materials

The following supporting information can be downloaded at: https://www.mdpi.com/article/10.3390/jcm15072698/s1, Table S1: Causes of graft lossin Sapienza Rome and Zagreb series; Table S2: Comparative analysis between patients with and without graft lossin the two cohorts.
