# Supplementary material for: Peak Lactate During the First Postoperative Day Predicts 90-Day Graft Loss After Liver Transplantation
Source: J Clin Med. 2026 Apr 2;15(7):2698. doi: 10.3390/jcm15072698 (PMC13073519; doi:10.3390/jcm15072698)
Supplement: Supplementary file 1 [file jcm-15-02698-s001.zip › jcm-4207243-supplementary.pdf]

Supplementary Table S1. Causes of graft loss in Sapienza Rome and Zagreb series.

Variables	Sapienza Rome (n=178)	Zagreb (n=90)
	N (%)	
Total n graft losses	46 (25.8)	17 (18.9)
Re-LT	4 (2.2) (HAT=1, IC=3)	5 (5.6) (HAT=1, PNF/PDF=3, IC=1)
Death due to		
Infection	17 (9.6)	7 (7.8)
PNF/PDF	8 (4.5)	1 (1.1)
Cerebral event	2 (1.1)	1 (1.1)
Pulmonary event	1 (0.6)	-
Cardiac event	1 (0.6)	1 (1.1)
Tumor recurrence	5 (2.8)	-
Cirrhosis recurrence	2 (1.1)	-
IC	2 (1.1)	-
De novo tumor	2 (1.1)	2 (2.2)
Other	2 (1.1)	-
90-day graft losses	25 (14.0)	10 (11.1)
Re-LT	2 (1.1) (HAT=1, IC=1)	3 (3.3) (HAT=1, PNF/PDF=2)
Death due to		
Infection	12 (6.7)	6 (6.7)
PNF/PDF	8 (4.5)	1 (1.1)
Cerebral event	2 (1.1)	-
Pulmonary event	1 (0.6)	-

Abbreviations: Re-LT, retransplantation; HAT, hepatic artery thrombosis; IC, ischemic cholangiopathy; PNF, primary non-function; PDF, primary dysfunction.

Supplementary Table S2. Comparative analysis between patients with and without graft loss in the two cohorts.

Variables	Sapienza Rome (n=178)		P	Zagreb (n=90)		P
	**No 90-day graft loss (n=153, 86.0%)**	**90-day graft loss (n=25, 14.0%)**		**No 90-day graft loss (n=80, 88.9%)**	**90-day graft loss (n=10, 11.1%)**	
	**Median (Q1-Q3) or n (%)**			**Median (Q1-Q3) or n (%)**		
**Patient-related variables**						
Age, years	57.0 (50.0-62.0)	51.0 (47.0-59.0)	0.01	59.5 (56.0-65.0)	67.5 (54.2-68.8)	0.29
Male sex	130 (85.0)	20 (80.0)	0.56	62 (77.5)	7 (70.0)	0.69
Caucasian	148 (96.7)	24 (96.0)	1.00	80 (100.0)	10 (100.0)	1.00
BMI	27.0 (24.0-29.0)	25.0 (23.0-29.0)	0.61	26.5 (23.0-29.0)	25.5 (21.8-27.5)	0.27
WL duration, months	5.1 (1.4-9.4)	0.3 (0.1-5.2)	<0.001	2.8 (0.9-5.6)	0.2 (0.0-1.6)	0.004
HCC positivity	82 (53.6)	5 (20.0)	0.002	30 (37.5)	2 (20.0)	0.49
HCV-related cirrhosis*	52 (34.0)	5 (20.0)	0.17	4 (5.0)	0 (0.0)	1.00
HBV-related cirrhosis*	27 (17.6)	5 (20.0)	0.78	5 (6.2)	0 (0.0)	1.00
Alcohol-related cirrhosis*	65 (42.5)	8 (32.0)	0.32	45 (56.2)	4 (40.0)	0.503
MASLD-related cirrhosis*	25 (16.3)	1 (4.0)	0.13	2 (2.5)	0 (0.0)	1.00
ALF*	8 (5.2)	12 (48.0)	<0.001	1 (1.2)	1 (10.0)	0.21
Other disease as indication for LT*	14 (9.2)	1 (4.0)	0.70	12 (15.0)	3 (30.0)	0.36
MELD	15.0 (10.0-22.0)	28.0 (21.0-31.0)	<0.001	17.0 (10.8-25.2)	18.5 (13.5-31.8)	0.59
MELDNa	16.0 (9.0-22.0)	27.0 (21.0-33.0)	<0.001	20.5 (12.0-29.2)	20.0 (15.0-33.5)	0.74
**Transplantation-related variables**						
CIT, minutes	400.3 (395.0-428.3)	400.3 (356.0-400.3)	0.08	262.0 (219.0-356.0)	390.0 (283.0-443.0)	0.09
WIT, minutes	63.4 (57.0-67.0)	64.2 (62.5-72.8)	0.09	46.0 (39.0-63.0)	50.0 (40.8-73.2)	0.66
Lactates LT declamping, mmol/L	3.5 (2.8-3.9)	3.8 (3.2-4.8)	0.12	4.3 (3.4-5.0)	4.3 (4.0-4.9)	0.67
Lactates end of LT, mmol/L	2.4 (1.7-3.4)	2.8 (2.1-3.4)	0.19	1.6 (1.0-2.7)	3.3 (1.9-5.0)	0.02
Lactates at 1 day after LT, mmol/L	1.5 (1.1-2.0)	1.7 (1.4-2.0)	0.07	11.0 (0.8-15.0)	17.0 (14.5-33.2)	0.008
Lactates peak within post-LT Day 1, mmol/L	3.5 (2.8-4.3)	8.4 (5.7-8.4)	<0.001	4.4 (3.4-5.1)	7.7 (5.1-15.3)	0.003
AST peak within post-LT Day 3, IU/L	865.0 (495.0-1373.0)	897.0 (436.0-1708.0)	0.83	829.5 (506.0-1434.5)	2126.0 (821.2-4567.0)	0.06
ALT peak within post-LT Day 3, IU/L	595.0 (355.0-1133.0)	507.0 (405.0-1640.0)	0.54	526.5 (263.2-906.0)	1502.5 (415.2-2373.8)	0.10
Bilirubin peak within post-LT Day 3, mg/dL	3.3 (2.0-6.2)	5.4 (2.9-7.6)	0.054	0.6 (0.4-1.1)	1.9 (1.1-1.9)	0.008
INR peak within post-LT Day 3	1.4 (1.3-1.6)	1.5 (1.4-1.6)	0.047	1.5 (1.3-1.7)	2.0 (1.3-2.3)	0.08
Bilirubin on post-LT Day 7, mg/dL	6.8 (3.5-11.0)	9.6 (4.9-22.4)	0.001	0.5 (0.3-0.9)	1.4 (0.8-1.9)	0.008
INR on post-LT Day 7	1.2 (1.1-1.3)	1.3 (1.2-1.5)	0.002	1.1 (1.0-1.1)	1.2 (1.1-1.3)	0.10
EAD	54 (35.3)	12 (48.0)	0.22	12 (15.0)	6 (60.0)	0.004
MEAF	4.3 (3.0-5.5)	4.4 (3.7-6.4)	0.20	2.7 (1.5-3.6)	5.0 (3.2-6.4)	0.02
MEAF ≥5	51 (33.3)	12 (48.0)	0.16	7 (8.8)	5 (50.0)	0.003
ICU stay, days	7.0 (5.0-10.0)	34.0 (11.0-69.0)	<0.001	3.0 (2.0-4.0)	3.5 (3.0-17.5)	0.057
Length of stay, days	18.0 (16.0-29.0)	39.0 (17.0-72.0)	0.005	14.0 (10.8-23.8)	33.5 (22.2-42.5)	0.058
Clavien-Dindo ≥3a	28 (18.3)	24 (96.0)	<0.001	37 (46.2)	10 (100.0)	0.001
**Donor-related variables**						
Age, years	57.0 (43.0-67.0)	60.0 (49.0-71.0)	0.38	63.5 (52.8-71.0)	58.5 (54.2-66.5)	0.50
Male sex	78 (51.0)	12 (48.0)	0.78	48 (60.0)	2 (20.0)	0.02
Trauma as cause of death	36 (23.5)	9 (36.0)	0.18	26 (32.5)	1 (10.0)	0.27
Anoxia as cause of death	2 (1.3)	1 (4.0)	0.37	6 (7.5)	0 (0.0)	1.00
Cerebrovascular accident as cause of death	110 (71.9)	15 (60.0)	0.23	49 (61.2)	9 (90.0)	0.09
Other condition as cause of death	1 (0.7)	0 (0.0)	1.00	5 (6.2)	0 (0.0)	1.00
ICU stay, days	4.0 (2.0-5.0)	4.0 (3.0-6.0)	0.40	5.0 (3.0-8.0)	2.5 (2.0-3.8)	0.009
BMI	26.0 (24.0-28.0)	25.0 (24.0-27.0)	0.57	28.0 (24.8-31.0)	28.5 (24.0-29.8)	0.99

* Some patients presented multiple causes of liver disease contemporaneously.

Variables: Q1, 25% quartile; Q3, 75% quartile; BMI, body mass index; WL, waiting list; HCC, hepatocellular carcinoma; HCV, hepatitis C virus; HBV, hepatitis B virus; MASLD, metabolic dysfunction-associated steatotic liver disease; ALF, acute liver failure; LT, liver transplantation: MELD, model for end-stage liver disease; Ma, sodium; CIT, cold ischemia time; WIT, warm ischemia time; AST, aspartate aminotransferase; ALT, alanine aminotransferase; INR, international normalized ratio; EAD, early allograft dysfunction; ICU, intensive care unit.
